# Quorum Quenching Properties and Probiotic Potentials of Intestinal Associated Bacteria in Asian Sea Bass *Lates calcarifer*

**DOI:** 10.3390/md18010023

**Published:** 2019-12-26

**Authors:** Reza Ghanei-Motlagh, Takavar Mohammadian, Darioush Gharibi, Simon Menanteau-Ledouble, Esmaeil Mahmoudi, Mohammad Khosravi, Mojtaba Zarea, Mansour El-Matbouli

**Affiliations:** 1 Clinical Division of Fish Medicine, University of Veterinary Medicine, 1210 Vienna, Austria; reza.ghanei@vetmeduni.ac.at (R.G.-M.); matboulim@staff.vetmeduni.ac.at (M.E.-M.); 2 Department of Clinical Sciences, Faculty of Veterinary Medicine, Shahid Chamran University of Ahvaz, Ahvaz 61357-831351, Iran; moj.zarea@gmail.com; 3 Department of Pathobiology, Faculty of Veterinary Medicine, Shahid Chamran University of Ahvaz, Ahvaz 61357-831351, Iran; d.gharibi@scu.ac.ir (D.G.); m.khosravi@scu.ac.ir (M.K.); 4 Department of Plant Protection, Faculty of Agriculture, Isfahan (Khorasgan) Branch, Islamic Azad University, Isfahan 158-81595, Iran; e.mahmoudi@khuisf.ac.ir

**Keywords:** *Lates calcalifer*, vibriosis, quorum quenching, probiotic

## Abstract

Quorum quenching (QQ), the enzymatic degradation of *N*-acyl homoserine lactones (AHLs), has been suggested as a promising strategy to control bacterial diseases. In this study, 10 AHL-degrading bacteria isolated from the intestine of barramundi were identified by 16S rDNA sequencing. They were able to degrade both short and long-chain AHLs associated with several pathogenic *Vibrio* species (spp.) in fish, including *N*-[(RS)-3-Hydroxybutyryl]-l-homoserine lactone (3-oh-C_4_-HSL), *N*-Hexanoyl-l-homoserine lactone (C_6_-HSL), *N*-(β-Ketocaproyl)-l-homoserine lactone (3-oxo-C_6_-HSL), *N*-(3-Oxodecanoyl)-l-homoserine lactone (3-oxo-C_10_-HSL), *N*-(3-Oxotetradecanoyl)-l-homoserine lactone (3-oxo-C_14_-HSL). Five QQ isolates (QQIs) belonging to the *Bacillus* and *Shewanella* genera, showed high capacity to degrade both synthetic AHLs as well as natural AHLs produced by *Vibrio harveyi* and *Vibrio alginolyticus* using the well-diffusion method and thin-layer chromatography (TLC). The genes responsible for QQ activity, including *aiiA*, *ytnP,* and *aaC* were also detected. Analysis of the amino acid sequences from the predicted lactonases revealed the presence of the conserved motif HxHxDH. The selected isolates were further characterized in terms of their probiotic potentials in vitro. Based on our scoring system, *Bacillus thuringiensis* QQ1 and *Bacillus cereus* QQ2 exhibited suitable probiotic characteristics, including the production of spore and exoenzymes, resistance to bile salts and pH, high potential to adhere on mucus, appropriate growth abilities, safety to barramundi, and sensitivity to antibiotics. These isolates, therefore, constitute new QQ probiotics that could be used to control vibriosis in *Lates calcalifer*.

## 1. Introduction

The rapid development of aquaculture as one of the main sectors for the production of high quality and bioavailable protein has led to the intensification of rearing systems to meet the increasing demands for human consumption [1]. However, in intensive farming situations, several problems such as stressors and disease outbreaks are known to occur [2]. Bacterial diseases, particularly, those caused by gram-negative bacteria are considered as the major cause of mortalities in aquaculture [3]. Among the gram-negative bacteria, *Vibrio* species are well-known endemic bacteria associated with marine ecosystems, commonly found in tropical and temperate coastal and estuarine regions [4]. Apart from being pathogenic for aquatic animals, some species are responsible for several human infections [5]. The major infection of marine fish and shellfish, vibriosis, is frequently associated with stress conditions contributing to severe losses, whereas pathogenicity in some strains is independent of any predisposing stress [6]. The control of bacterial diseases such as vibriosis is commonly achieved through antibiotic-therapy. However, inappropriate application of antibiotics can lead to antibiotic residues in different tissues as well as imbalances within the gut microflora of treated fish as well as to the emergence of antibiotic resistance [7]. Further obstacles associated with the use of antibacterial agents are their high cost, inefficacy in off-feed fish, environmental side-effects, and ineffectiveness against biofilm-producing bacteria [8,9,10]. Unfortunately, contrary to the increased rate of antibacterial resistances, the discoveries of newly alternative antibiotics have severely declined in the last several decades [11]. Therefore, in order to maintain eco-friendly and sustainable aquaculture, there is a requirement to develop novel alternatives for disease prophylaxis and treatment [7].

Use of beneficial microorganisms targeting communication systems (quorum sensing) of pathogens rather than their fundamental structures or processes, is an attractive strategy to control conventional and emerging infections [12]. Quorum sensing (QS) is a process by which bacteria monitor their population in a cell-density dependent manner through the synthesis, exchange, and the detection of small intracellular signals (autoinducers) [13]. Among the various types of signals that are produced in bacteria (Autoinducer-1, Autoinducer-2, and Autoinducing peptides), Autoinducer-1 or AHLs generated by gram-negative bacteria are probably the best studied. Bacteria produce the QS molecules, and when the intensity of these signals reaches a threshold, they diffuse back into the bacterial cell and regulate the expression of QS-related genes such as those involved in biofilm formation and production of virulence determinants [14]. Indeed, QS allows bacteria to operate as multicellular organisms. Since AHL-based QS is common among marine *Vibrio*, improving our understanding of the procedures related to QS generation by Vibrios and techniques to disrupt these mechanisms will provide new eco-friendly methods for the prevention and treatment of *Vibrio* infections [15]. QQ is a phenomenon opposite to QS and refers to the inactivation of the chemical signals via a mechanism mediated by a number of enzymes, including AHL lactonase, AHL acylase, and AHL oxidoreductase [11]. Signal degradation has been shown to hinder certain important QS-associated phenotypes such as virulence factors and biofilm development but not growth; hence this allows the host defense mechanisms to interfere with the bacterial infection. As an anti-infective strategy, QQ may also compel less selective pressure on pathogens, and in turn, delay the development of resistance mechanisms [16].

Application of AHL degrading bacteria able to utilize AHL molecules of marine and freshwater pathogens as a food source is a developing idea due to their previously successful outcomes [17,18,19,20,21,22]. However, the majority of the strains that have been tested in the past are allochthonous QQ bacteria [21,23]. Moreover, the isolation of autochthonous bacteria with QQ potential has frequently been reported from freshwater fish [19,24,25,26], and to our knowledge, there is only one report in marine fish [27]. On the other hand, despite the high mortalities caused by marine vibriosis, QQ strategy has not been adopted against commonly occurring *Vibrio* pathogens in fish, particularly, *L. calcarifer*, a euryhaline marine fish with high economic importance that has gained much attention from researchers and farmers in the last decade. In contrast with some probiotics that are able to out-compete pathogens through the production of antimicrobials, QQ probiotics are neither bactericidal nor bacteriostatic against the targeted pathogens. Instead, they disrupt QS signals and affect the pathogenicity of their target. On the contrary, one of the main criteria for screening potential probiotic candidates is the inhibitory activity against different pathogens [28]. Since the transit of probiotics through the gastrointestinal tract may detrimentally affect their functionality and viability, candidate probiotic bacteria must be resistant to the acidic pH of the stomach, bile salts, and digestive enzymes to reach the intestine, colonize the host, and finally activate their beneficial effects [29]. However, there is no comprehensive investigation of the real probiotic characterization of QQ bacteria prior to their administration to fish and their application on a commercial scale. Thus, screening for strains that not only exhibit QQ capacity but also have appropriate probiotic hallmarks would be of interest. In the present study, QQ bacteria with a potential to degrade the dominant range of AHL molecules produced by several significant and prevalent pathogenic *Vibrio* spp. in fish, were isolated from the intestine of barramundi and characterized and their efficacy as autochthonous probiotics was tested for the first time.

## 2. Results

### 2.1. Screening for AHL Degrading Bacteria

Following purification, 88 bacterial isolates representing different colony morphologies were obtained from the intestine of barramundi fish. They were analyzed for AHL degradation activity using minimal agar plates, containing AHL as the sole energy source. Seventeen isolates were selected based on their ability to grow on minimal media. These isolates were additionally screened by streaking alongside two biosensor strains, *Chromobacterium violaceum* CV026 and *Agrobacterium tumefaciens* NTL4 (pZLR4). Seven isolates that secreted their own QS molecules, as shown by the apparition of blue or purple color in the reporter strains, were excluded. Finally, 10 QQIs with no activity for the production of AHLs were selected for further study.

### 2.2. Calibration Curve and Kinetics of AHL Degradation

**Figure 1:**
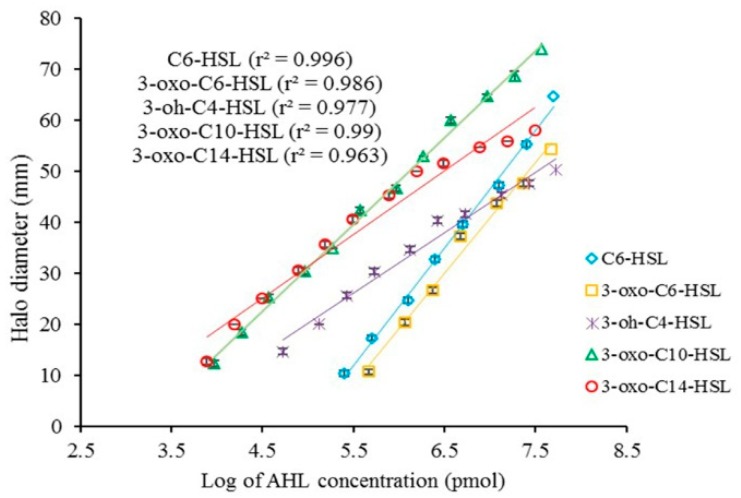
Calibration curves between the doses of *N*-acyl homoserine lactones (AHLs) and the halo diameter generated by the biosensors (mean ± SD, *n* = 3).

**Figure 2:**
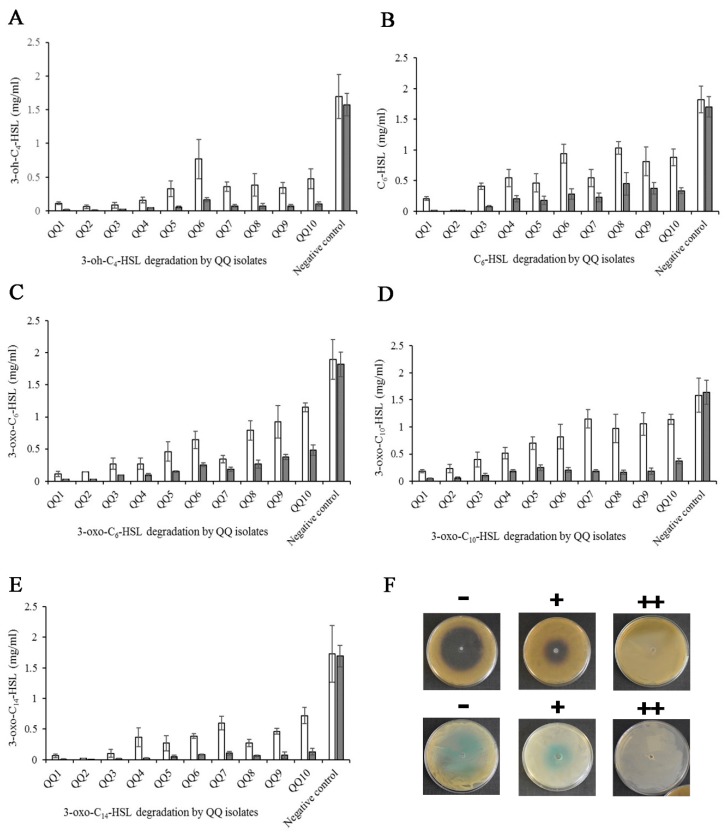
Degradation of synthetic AHLs. (**A**–**E**) Kinetics of AHL degradation by the selected strains. Measurements were taken at 24 (white bars) and 48 h (grey bars) intervals (mean ± SD, *n* = 3). (**F**) QQ activity detected by *C. violaceum* CV026 and *A. tumefaciens* NTL4: no AHL degradation (−), imperfect AHL degradation (+), and perfect AHL degradation (++).

The concentration of AHLs directly correlated with the size of the colored halos in the biosensors. Calibration (standard) curves for different AHLs were obtained by plotting the log of various concentrations of AHLs (pmol) versus the size of the induced zones (Figure 1). The standard curves were subsequently used to estimate the kinetic of AHL degradation. The size of the halos was converted to the log of concentrations of AHLs, followed by the antilog of the obtained data. Finally, a new standard curve related to the conversion of the concentration units (pmol to mg/L) of AHLs was constructed (Figure 2).

The potential of the isolates varied in terms of the degradation of AHLs. In total, the isolates QQ1, QQ2, QQ3, QQ4, and QQ5 showed a high degrading ability for all tested AHLs (*P* < 0.05). The concentration of AHLs in almost all the QQIs was below 0.5 mg/L at 48 h after incubation. Among the tested AHLs, 3-oxo-C_10_-HSL was more resistant to deactivation by the QQIs. The concentration of AHLs in the negative controls, Luria–Bertani (LB) broth supplemented with AHLs, was stable after 48 h incubation, suggesting no chemical degradation of the signals during the experiments.

### 2.3. Identification of QQ Bacteria

The 16S rDNA sequence analysis of the QQIs showed that they belonged to the *Bacillus*, *Shewanella* and *Carnobacterium* genera (Table 1). *Bacillus* species were the most frequent in the intestine, representing eight out of the 10 QQIs identified. The identified sequences were deposited at the Genebank database with an accession number (Table 1).

### 2.4. Sequence Alignment and Phylogenetic Analyses

**Figure 3:**
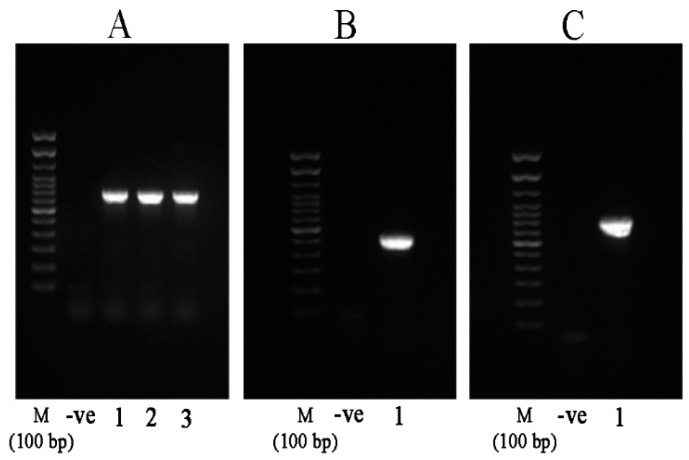
Agarose gel electrophoresis showing the PCR-amplified products from the Quorum quenching (QQ) genes. (**A**) Amplification of the AiiA gene from *B. thuringiensis* QQ1 (lane 1), *B. cereus* QQ2 (lane 2), and *B. thuringiensis* QQ3 (lane 3) with a product size ~750 bp. (**B**) Partial amplification of the YtnP gene from *Bacillus* sp. QQ4 (lane 1, expected amplicon size ~530 bp). (**C**) Partial amplification of the acylase gene homolog from *S. algae* (PCR product size ~820 bp, lane 1). M stands for marker and -ve for negative control.

**Figure 4:**
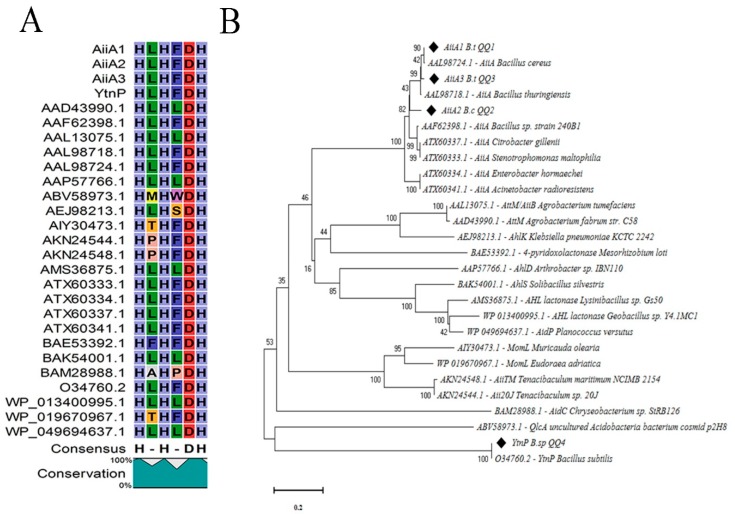
Comparison of the amino acid sequences of AHL lactonase enzymes. (**A**) Sequences corresponding to lactonase AiiA 1–3 (from QQ1, QQ2, QQ3) and YtnP (from QQ4) were compared with other known AHL lactonases from the MBL fold metallohydrolase superfamily, and all represented the common conserved motif ‘HxHxDH’. Each part of this motif is represented by a specific letter and color. Columns that are of the same color and with the same letter show motifs conserved between all sequences. (**B**) Phylogenetic analysis based upon amino acid sequences of detected AHL lactonases (AiiA and YtnP, black quadrates) and the homolog sequences, including AiiA from *Bacillus thuringiensis* (AAL98718.1), AiiA from *Bacillus cereus* (AAL98724.1), AiiA from *Bacillus* sp. strain 240B1 (AAF62398.1), AttM/AiiB from *Agrobacterium tumefaciens* (AAL13075.1), AhlD from *Arthrobacter* sp. IBN110 (AAP57766.1), QlcA from uncultured *Acidobacteria* bacterium cosmid p2H8 (ABV58973.1), AhlK from *Klebsiella pneumoniae* KCTC 2242 (AEJ98213.1), MomL from *Muricauda olearia* (AIY30473.1), MomL from *Eudoraea adriatica* (WP_019670967.1), AidC from *Chryseobacterium* sp. StRB126 (BAM28988.1), 4-pyridoxolactonase from *Mesorhizobium loti* (BAE53392.1), AhlS from *Solibacillus silvestris* (BAK54001.1), AHL lactonase from *Lysinibacillus* sp. Gs50 (AMS36875.1), AHL lactonase from *Geobacillus* sp. Y4.1MC1 (WP_013400995.1), AiiA from *Enterobacter hormaechei* (ATX60334.1), AiiA from *Acinetobacter radioresistens* (ATX60341.1), AiiA from *Citrobacter gillenii* (ATX60337.1), AiiA from *Stenotrophomonas maltophilia* (ATX60333.1), AttM from *Agrobacterium fabrum* str. C58 (AAD43990.1), AiiTM from *Tenacibaculum maritimum* NCIMB 2154 (AKN24548.1), Aii20J from *Tenacibaculum* sp. 20J (AKN24544.1), and AidP from *Planococcus versutus* (WP_049694637.1). The dendrogram was generated by the neighbor-joining method using the MEGA X software. The scale bar represents 0.2 substitutions per amino acid position.

**Figure 5:**
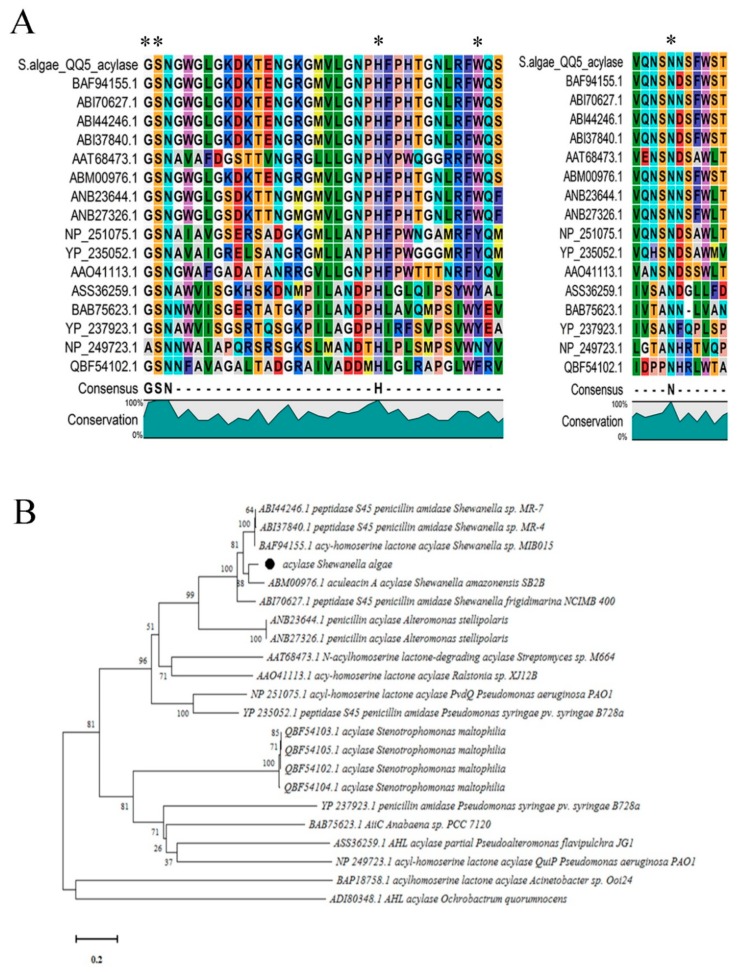
Comparison of the amino acid sequences of quorum quenching acylase enzymes. (**A**) The comparison of amino acid sequences of putative *N*-acyl homoserine lactones (AHLs) acylase (from QQ5) with other known AHL acylases from the Ntn hydrolase superfamily represented the important residues for the catalytic processing in known acylases (shown by asterisks). Each part of this motif is represented by a specific letter and color. Columns that are of the same color and with the same letter show motifs conserved between all sequences. (**B**) Phylogenetic tree based on amino acid sequences resulting from the acylase of the *Shewanella algae* (Black circle) and other known acylases from *Stenotrophomonas maltophilia* (QBF54102.1, QBF54103.1, QBF54104.1, and QBF54105.1), *Acinetobacter* sp. Ooi24 (BAP18758.1), *Ochrobactrum quorumnocens* (ADI80348.1), *Streptomyces* sp. M664 (AAT68473.1), *Pseudomonas aeruginosa* PAO1 (NP_251075.1 and NP_249723.1), *Shewanella* sp. MIB015 (BAF94155.1), *Alteromonas stellipolaris* (ANB23644.1 and ANB27326.1), *Pseudoalteromonas flavipulchra* JG1 (ASS36259.1), *Ralstonia* sp. XJ12B (AAO41113.1), *Anabaena* sp. PCC 7120 (BAB75623.1), *Shewanella* sp. MR-7 (ABI44246.1), *Shewanella* sp. MR-4 (ABI37840.1), *Shewanella frigidimarina* NCIMB 400 (ABI70627.1), *Shewanella amazonensis* SB2B (ABM00976.1) and *Pseudomonas syringae* pv. *syringae* B728a (YP_235052.1 and YP_237923.1). The tree was constructed by the neighbor-joining method using the MEGA X software. The scale bar represents 0.2 substitutions per amino acid position.

We chose the strains QQ1, QQ2, QQ3, QQ4, and QQ5 to continue the study as these were the isolates that showed the most potent QQ activity. We confirmed the presence of genes associated with AHLs degradation in the selected QQIs: homologues of the *aiiA* gene were detected in *Bacillus* strains QQ1, QQ2, and QQ3, *ytnP* gene in the *Bacillus* sp. QQ4 and *aaC* gene in the *Shewanella algae* QQ5 (Figure 3). Subsequently, the BLAST analysis of the sequences obtained (accession numbers SAMN13290781 to SAMN13290785) confirmed the close identity rates of the sequences QQ1, QQ2, and QQ3 to *aiiA* lactonase from the multispecies of the *Bacillus* genus (91–100%), QQ4 to *ytnP* lactonase from the *Bacillus subtilis* (99.37%), and QQ5 to AHL acylase from *Shewanella* sp. MIB015 (85.66%). The multiple alignment analysis of the amino acid sequences from the predicted lactonases with those from the known AHL lactonases in the metallo-β-lactamase superfamily revealed the presence of the commonly conserved zinc-binding motif HxHxDH (Figure 4A) and two metal-binding sites with high Histidine (H) and Aspartic acid (A). AHL lactonases from the isolates QQ1, QQ2, and QQ3 were found to be in the same group as the respective representatives of the AiiA group. Putative AHL lactonase YtnP from the strain QQ4 was located in a separate cluster with QlcA (Figure 4B). Analysis of amino acid sequences from putative AHL acylase QQ5 with those from the known AHL acylase in the N-terminal nucleophile amidohydrolase (Ntn hydrolases) superfamily indicated the presence of amino acid residues involved in substrate specificity (Figure 5A). The putative AHL acylase from *S. algae* was phylogenetically included in the acylases belonging to the *Shewanella* group (Figure 5B).

### 2.5. Antimicrobial Activity of QQIs

Prior to assessing the ability of QQIs to degrade natural AHLs extracted from *V. harveyi* and *V. alginolyticus*, their antagonistic ability against these pathogens was investigated. None of the QQIs exerted direct or extracellular antibacterial activity towards to *Vibrio* spp. tested, thereby confirming that they could be used as probiotics to disturb the QS system without any negative effect on the growth of the pathogens.

### 2.6. AHL Profiling and Degradation of V. harveyi and V. alginolyticus

**Figure 6:**
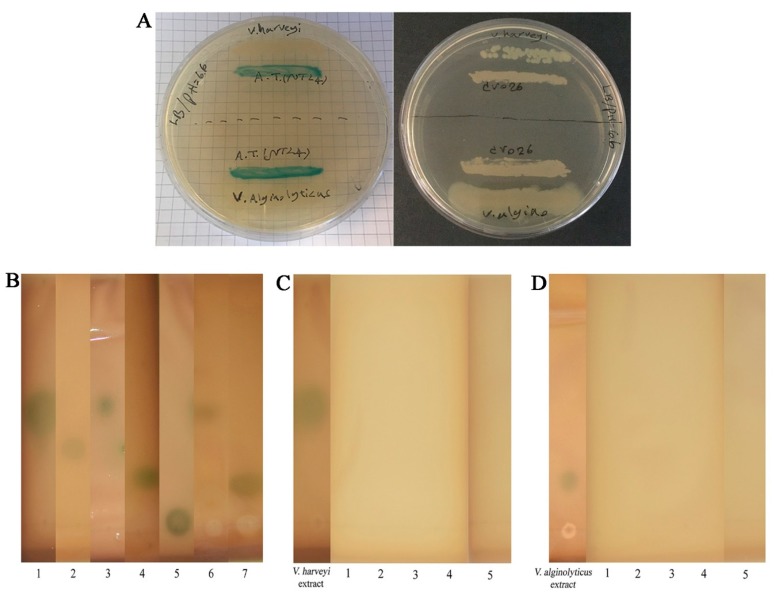
Detection and degradation of natural AHLs. (**A**) Detection of AHLs produced by tested *V. harveyi* and *V. alginolyticus* by the cross-feeding assay. Induction of *A. tumefaciens* NTL4 (left plate) vs. non-induction of *C. violaceum* CV026 (right plate). (**B**) Thin-layer chromatography (TLC) analysis for tentative identification of AHLs extracted from tested *V. harveyi* (lane 6) and *V. alginolyticus* (lane 7). Synthetic AHLs were used as standards. Lane 1 (3-oh-C_4_-HSL), lane 2 (C_6_-HSL), lane 3 (3-oxo-C_6_-HSL), lane 4 (3-oxo-C_10_-HSL), and lane 5 (3-oxo-C_14_-HSL). (**C**–**D**) Visualization of the AHL-degrading activity of selected QQIs against natural AHLs of *V. harveyi* and *V. alginolyticus* using *A. tumefaciens* NTL4 overlay on TLC plates. The absence of blue color development in *A. tumefaciens* NTL4 indicates positive QQ activity. AHLs extracted from *V. harveyi* and *V. alginolyticus* cultures were regarded as negative controls. QQ1 (lane 1), QQ2 (lane 2), QQ3 (lane 3), QQ4 (lane 4), QQ5 (lane 5).

The AHL profiles of the pathogenic bacteria studied, *V. harveyi* and *V. alginolyticus*, were analyzed using two biosensor systems. These methods (cross-feeding and the agar well-diffusion assay) have the advantage of rapid recognition of AHLs in a qualitative manner. Neither of the pathogens was able to induce the detector *C. violaceum* CV026 suggesting the absence of detectable short AHLs. On the other hand, the NTL4 strain of *A. tumefaciens* responded to the secreted AHLs of *V. alginolyticus*, indicating that this bacterium generated signals associated with medium or long-type AHLs. Detection of the singular signal of *V. harveyi* by *A. tumefaciens* NTL4 suggests the sensitivity of this detector to 3-oh-C_4_-HSL, apart from the medium to long AHLs (Figure 6A).

The crude extract AHL profiles of *V. harveyi* and *V. alginolyticus* was tentatively identified on thin-layer chromatography (TLC) plates overlaid with *A. tumefaciens* NTL4. The retention factors (R_f_) obtained for the developed color spots from *V. harveyi* (0.52) and *V. alginolyticus* (0.2) showed a high level of similarity with those of the standards 3-oh-C_4_-HSL (R_f_ = 0.55), C_6_-HSL (R_f_ = 0.43), 3-oxo-C_6_-HSL (R_f_ = 0.58), 3-oxo-C_10_-HSL (R_f_ = 0.21), and 3-oxo-C_14_-HSL (R_f_ = 0.08). This suggested a close similarity between the signals of the *V. alginolyticus* tested with that of the 3-oxo-C_10_-HSL and *V. harveyi* with 3-oh-C_4_-HSL (Figure 6B). To ascertain the quenching capacity of the selected isolates toward natural AHLs directly extracted from the tested vibrio cultures, we analyzed the residues of the signals by the agar well-diffusion assay, as well as TLC plates overlaid with *A. tumefaciens* NTL4. Following incubation of the extracted signals with bacterial cultures for 24 h, all QQIs inactivated the signals derived from tested *V. harveyi* and *V. alginolyticus* (Figure 6C,D).

### 2.7. Localization of the QQ Enzymes

AHL degrading activity from the supernatants of selected QQIs was checked, and no degradation activity was detected, demonstrating the cell-bound location of the respective enzymes.

### 2.8. Extracellular Enzymes and Sporulation

The abilities of selected isolates to produce exoenzymes and spores are reported in Table 2. Protease activity was observed in all isolates with higher activity in the isolates QQ2, QQ4, and QQ5. Lipase activity was detected in 4 isolates QQ1, QQ2, QQ4, and QQ5. QQ1 and QQ4 showed the greatest lipase activity. Moderate amylase activity was found in all *B. thuringiensis* strains. Phytase activity was identified in QQ3 and QQ4 isolates. All *Bacillus* spp. had the capability for sporulation (Table 2).

### 2.9. Resistance to Bile Salts and pH

The viability of the isolates in different concentrations of bile salts was recorded in Supplementary Table S1. All strains survived in bile salts ranging from 2.5 to 7.5%. Bacterial counts were significantly decreased in *B. thuringiensis* QQ3 at all levels of bile (*P* < 0.05). All studied *Bacillus* strains were tolerant to excessive acidity during 1.5 h incubation (see Supplementary Table S2). *Bacillus* isolates QQ1, QQ2, and QQ4 were more resistant to both alkaline and acidic pH conditions when compared to the other isolates. No viability of *S. algae* QQ5 was observed at very low pH ranges.

### 2.10. Tolerance to Sodium Chloride Salt

There were significant differences between the strains in terms of growth in various salinities (*P* < 0.05). Overall, higher growth in tryptic soy broth (TSB) media supplemented with 0–4% NaCl was observed in isolates QQ1, QQ2, and QQ5 (Supplementary Table S3).

### 2.11. Cell Surface Hydrophobicity

**Figure 7:**
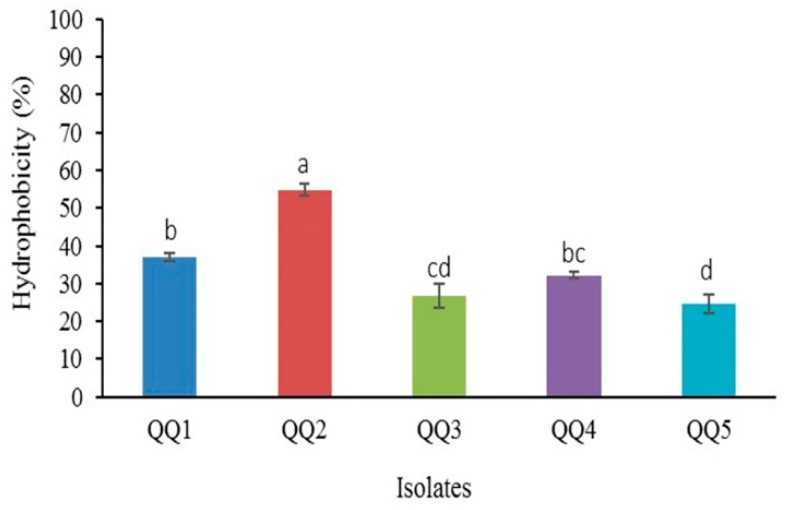
Hydrophobicity percentage of the quorum quenching bacteria (Mean ± SD, *n* = 3). Dissimilar letters show a significant difference among the isolates (*P* < 0.05). Means were rated as a (4), ab (3.5), b (3), bc (2.5), c (2), cd (1.5), d (1). The total score for the isolates was recorded as follows: QQ1 (3), QQ2 (4), QQ3 (1.5), QQ4 (2.5), QQ5 (1).

The percentage of hydrophobicity of the strains ranged from 24.57% to 54.85% (Figure 7). The percent in *Bacillus* isolates QQ1 and QQ2 was significantly higher than that of the other tested strains (*P* < 0.05).

### 2.12. Aggregation Ability

**Figure 8:**
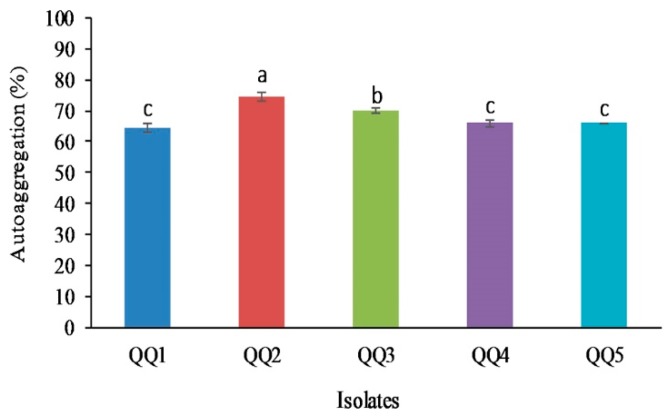
The percentage of auto-aggregation in quorum quenching bacteria (Mean ± SD, *n* = 3). Different alphabets represent a significant difference between isolates (*P* < 0.05). The rating of means was performed as follows a (4), b (3), c (2). The total score of each isolate was QQ1 (2), QQ2 (4), QQ3 (3), QQ4 (2), QQ5 (2).

The co-aggregation ability of the selected strains with *V. harveyi* and *V. alginolyticus* is summarized in the Supplementary Table S4. The maximal percentage of the co-aggregation with *V. harveyi* was observed after 2 h in QQ2 isolate and after 24 h in isolate QQ1 (*P* < 0.05). Co-aggregation with *V. alginolyticus* was significantly higher in isolate QQ2 than the other isolates (*P* < 0.05). The auto-aggregation varied between the strains from 65.85% to 74.48% (Figure 8). *B. cereus* QQ2 showed higher potential for this characteristic, followed by *B. thuringiensis* QQ3 (*P* < 0.05).

### 2.13. Potential for Growth on Intestinal Mucus

As indicated in Supplementary Table S5, *S. algae* QQ5 showed the highest specific growth on mucus (*P* < 0.05). All strains had a higher growth rate in marine broth in comparison with mucus. Shorter doubling time in mucus and TSB medium was observed in *S. algae* QQ5 and *B. thuringiensis* QQ1, respectively (*P* < 0.05).

### 2.14. Adhesion to Intestinal Mucus

**Figure 9:**
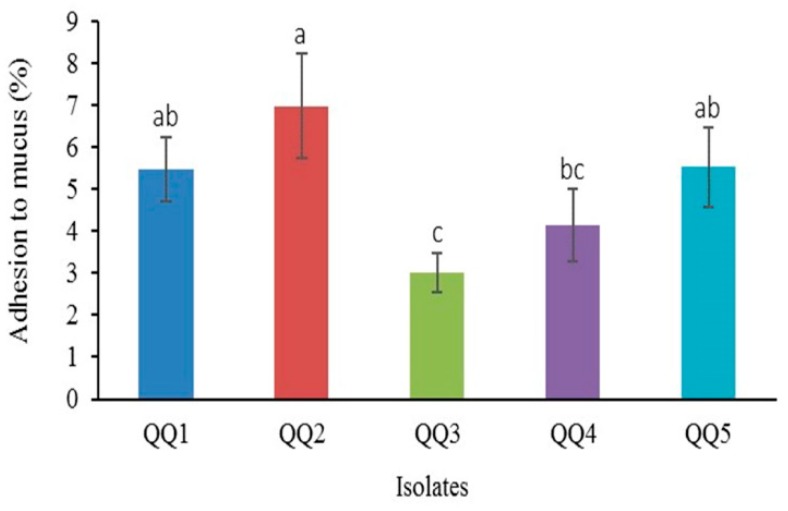
The adhesion potential of quorum quenching bacteria to intestinal mucus (Mean ± SD, *n* = 6). Different superscript letters denote significant differences between means (*P* < 0.05). Values were scored as a (4), ab (3.5), b (3), bc (2.5), c (2). The total score calculated for each isolate was the following: QQ1 (3.5), QQ2 (4), QQ3 (2), QQ4 (2.5), QQ5 (3.5).

The findings of the adhesion ability of the QQIs to mucus are represented in Figure 9. The highest and lowest ability for adherence to intestinal mucus was observed in *B. cereus* QQ2 (6.97%) and *B. thuringiensis* QQ3 (3%) (*P* < 0.05).

### 2.15. Antibiotic Susceptibility

All QQIs were susceptible to all tested antibiotics, with the exception of trimethoprim/sulfamethoxazole. The strains QQ1, QQ2, and QQ3 were resistance to this antibiotic (Supplementary Table S6).

### 2.16. Adverse Effects of QQIs in Barramundi Fish

No mortality and no moribund fish were observed following injection of fish with bacterial suspensions, and the bacteria could not be recovered from injected fish, indicating that the isolated strains had no pathogenic potential.

## 3. Discussion

Despite the effectiveness of vaccination and the widespread use of antibiotics to control vibriosis in aquaculture settings, anti-virulence therapy using compounds or organisms targeting the QS system hold great potential for the control of pathogens belonging to the *Vibrio* genus [1,17]. Among anti-infective therapies, the application of microorganisms that enzymatically inactivate AHL structures has proven to be useful in controlling infections of aquatic animals [30]. The marine ecosystem is an underexplored source of organisms or compounds with biological activity [31]. Given that the composition of gut microbiota is affected by the resources available, the bacteria-derived from the gastrointestinal tract could reflect those environmental bacteria able to colonize in this ecosystem [32].

In this study, several autochthonous AHL degrading bacteria were isolated from the gut of barramundi. They were able to partially or completely degrade the dominant synthetic signals produced by certain important and virulent pathogens related to marine vibriosis in fish, including *V. harveyi, V. campbelli, V. parahaemolyticus, V. alginolyticus, V. anguillarum* and *V. salmonicida*. According to ribotyping analysis, they belonged to three genera, including *Bacillus* (eight strains), *Shewanella* (one strain), and *Carnobacterium* (one strain). Consistently with our findings, *Bacillus* spp. originating from *Dicentrarchus labrax* and *Penaeus vannamei*, *Bacillus* QSI-1 from the intestinal tract of *Carassius auratus gibelio* as well as *B. cereus* and *B. thuringiensis* obtained from intestine of rainbow trout were isolated in the previous studies [18,24,26]. The 16S rDNA sequence analysis of *Bacillus* sp. QQ4 showed 99.76% similarity to *B. subtilis*, *B. velezensis* and *B. amyloliquefaciens*, an observation that is very similar to the findings of Zhou et al. (2018) [33]. Although *Bacillus* strains were already reported for QQ activity in various aquatic animals, here autochthonous *Bacillus* spp., able to degrade common AHLs belonging to *Vibrio* pathogens in fish, were isolated from the gastrointestinal tract of *L. calcalifer* for the first time. This study also constitutes the first report concerning the QQ potential in *S. algae* (QQ5). This is consistent with the work by Morohoshi et al. [34] who isolated a *Shewanella* sp. (MIB015) with AHL-degrading activity from the intestinal tract of Ayu fish (*Plecoglossus altivelis*) and suggested that this isolate could constitute an effective QQ autochthonous probiont [34]. Notably, these authors did not identify MIB015 at the species level.

We used minimal media supplemented with different synthetic AHLs as a sole source of carbon to isolate QQ bacteria, hence the bacteria able to grow on this medium possessed a possible type of enzyme involved in QQ activity. We investigated the presence of lactonase and acylase genes in 5 selected isolates (QQ1–QQ5). Autoinducer inactivation (*aiiA*) gene encoding AHL lactonase was detected in *Bacillus cereus* and *Bacillus thuringiensis* strains. The QQ activity in *Bacillus* sp. QQ4 seemed to be due to the presence of a putative QQ lactonase called *ytnP* encoding a lactonase homologous protein [35]. As most of AHL lactonases, both of the lactonases identified belonged to the metallo-β-lactamase superfamily. Several groups of this superfamily contain the consensus zinc-binding motif HxHxDH in the middle region [36,37,38]. As suggested by Thomas et al. (2005), completely conserved zinc-binding residues present in all known AHL lactonases are histidine 104, histidine 106, and histidine 169 in the metal site 1 and aspartic acid 108, histidine 109, and histidine 235 in the metal site 2 [39]. A mutagenic study of AiiA type lactonases highlighted the involvement of tyrosine (Y 194) and aspartic acid (D 108) residues in the catalytic mechanism [40]. The putative AHL acylase gene from *Shewanella algae* QQ5 was also amplified and sequenced. The BLAST analysis indicated that putative AHL acylase of *S. algae* QQ5 belonged to penicillin acylase family (aculeacin A acylase), which structurally appertains to the Ntn hydrolase superfamily [41]. It shared the identities between 35–85% with AHL acylases previously proven to irreversibly degrade the AHL structure (Aac, PvdQ, AiiC, and AhlM). As indicated by Morohoshi et al. (2008), the homologs of the AHL acylase in *Shewanella* spp. share conserved amino acid residues involved in their activity with other similar acylases; however, the tyrosine residues (Y) are replaced by tryptophan residues (W) at the position 295 [19].

*V. harveyi* produces a singular signal of AHL type, namely harveyi autoinducer 1 (HAI-1), whereas *V. alginolyticus* strains produce diverse kinds of AHLs dominantly including 3-oh-C_4_-HSL, 3-oxo-C_10_-HSL, and 3-oxo-C_14_-HSL [42,43]. By comparing the calculated R_f_ values on TLC plates, the studied *V. harveyi* and *V. alginolyticus* generated 3-oh-C_4_-HSL and 3-oxo-C_10_-HSL, respectively. The five selected bacteria able to produce QQ enzymes were tested in order to inactivate natural signals generated by two important fish pathogens, including *V. harveyi* and *V. alginolyticus* by the agar well-diffusion and TLC methods. All strains degraded the crude extracts containing natural short (3-oh-C_4_-HSL from *V. harveyi*) and long (3-oxo-C_10_-HSL from *V. alginolaticus*) side-chain AHLs, indicating their possible potential against the diseases that involve AHL-type autoinducers. Investigation of QQ activity in the cell-free supernatants of selected isolates showed that similar to most of lactonases and acylases previously described, cell-bound QQ enzymes were responsible for AHL degradation in these bacteria [12,24]. However, it has been reported that the QQ activity of some enzymes, such as HacB and AhlM, is secretory [16].

The involvement of the intestinal microflora in promoting health and modulating the immunity of fish is well documented, and modulation of the gut ecosystem using probiotics is increasingly adopted [22,44]. Selection of appropriate probionts requires in vitro preliminary tests, which assay the bacteria for the safety to host, sensitivity to antibiotics, tolerance to low pH and bile salts, their growth in and adherence to mucus, antagonistic properties against pathogens, production of relevant digestive exoenzymes, derivation from the host and favorable growth characteristics [45]. In the current study, the isolates selected were further screened by several in vitro probiotic tests, followed by the use of a rating system to achieve multifunctional probiotics with QQ benefits. It has been suggested that secondary metabolites synthesized by the fish microbiota might exert advantageous effects on the digestive processes of fish [46]. The tested probiotic candidates produced various extracellular hydrolytic enzymes with different potency, which enhance the digestion and nutrient uptake of available substrates. Previously, enzyme-producing bacteria, mainly *Bacillus* spp., have been isolated from the alimentary tracts of *Labeo rohita*, *Oreochromis mossambica,* and *Heteropneustes fossilis* [47,48,49]. *Bacillus* spp. have a high potential to produce functional enzymes that break down biological macromolecules, supply vitamins and fatty acids as well as degrade organic materials [33,46]. Thus, apart from endogenous digestive enzymes, there is a distinct microbial source of several exoenzymes in the intestine that might participate in and enhance the digestive process of aquatic animals. The colonization of probiotics in the host gut requires their passage through the stomach and anterior intestine, hence there is a tendency towards spore-forming bacteria that are likely to reach the distal intestine where spores persist and possibly germinate in higher number [44]. In this sense, *Bacillus* strains are widely used as probiotics in aquaculture because of their capability of spore formation [50]. Another important pre-requisite for successful passage is survival under gastric acid and high bile concentration [51]. In our study, all the *Bacillus* spp. were able to produce spore and withstand at different bile concentrations and pH conditions. However, the *Bacillus* strains QQ1, QQ2, and QQ4 were significantly more resistant to low pH and bile salts. The probiotic strains of *Bacillus* species were previously demonstrated to exhibit resistance to simulated gastrointestinal conditions that are attributable to their frequent isolation from the intestine of aquatic animals [52,53,54]. *Shewanella algae* QQ5 failed to survive at low pH; however, the effect of direct exposure of this strain with gastric acid and bile may not necessarily occur in vivo following incorporation to diet [55]. The optimum growth of *Bacillus* QQIs at 2% salinity, suggesting that they are suitable for use in marine water. The ability of the strain QQ5 for growth in various salinities could be explained by the fact that the sampling location from which this strain was isolated (Khuzestan) was a brackish water farming site with lower salinities (ranging between 5 to 18 ppt) compared to the other sampling sites. Moreover, the growth of all isolates at 0 to 8% NaCl may reflect their potential to be transferred and survive across freshwater and marine environments [56].

Another crucial criterion for the selection of probiotic bacteria is their potential to adhere to the mucosal surfaces of the intestine [57]. Adhesion is a sophisticated process involving specific ligand-receptor and non-specific (hydrophobicity) mechanisms. The characteristic of the bacterial cell surface (hydrophilic or hydrophobic nature) is a key factor in the adherence ability of bacteria to abiotic or biotic surfaces and biofilm formation [58]. In our work, the highest percentage of hydrophobicity was observed in *B. cereus* QQ2, suggesting the high potential of this strain to colonize the host. The difference in microbial cell hydrophobicity is associated mainly with proteins, carbohydrates (glycoproteins), and lipoteichoic acid on the cell surface [59]. Hydrophobicity could also affect the auto-aggregation of bacteria, and these two can act synergistically for attachment of a probiotic strain to epithelial surfaces of the host [60]. On the other hand, it is highly desirable for the probiotic candidates to be able to form multicellular aggregates associated with the same strain (auto-aggregation) or between two various strains (co-aggregation) [61]. In this study, *B. cereus* QQ2 manifested a good potential for auto-aggregation, as well as co-aggregation against *Vibrio* pathogens, suggesting the adhesion ability of QQ2 to intestinal mucosa and prevention of colonization by these pathogens. In congruence with our results, *Bacillus siamensis* strain B44v, *Bacillus subtilis* P33 and *Bacillus subtilis* P72 displayed useful probiotic properties including aggregation potential [54,57]. The ability of adhesion to and growth in mucus varied among QQ isolates. *B. cereus* QQ2 presented better adhesion ability to mucus that other QQIs. Higher growth rate and shorter doubling time in mucus were obtained for *S. algae* QQ5. As suggested by Geraylou et al. (2012), these differences may be owing to special nutrients or chemical composition included in mucus [62]. A short lag period and short-doubling time can result in the outcompeting of a probiotic to colonize the host and prevent infection [63]. Antibiotic sensitivity is another crucial requirement for candidate probiotics. We found the susceptibility of QQ isolates to all tested antibiotics except SXT to which the isolates QQ1, QQ2, and QQ3 showed resistance. *Bacillus* spp. were previously reported to be intrinsically resistant to β-lactam and sulfonamides antibiotics [64,65,66]. Furthermore, non-pathogenicity of candidate probiotics is required to ensure their safety in the host. Following injection, it was found that none of the QQ probiotics had harmful effects, indicating their potential for administration in aquaculture. We used a scoring system to rate candidate QQ isolates according to their probiotic characteristics. Considering the total score (102), *B. thuringiensis* QQ1 achieved the highest score (84.5), followed by *B. cereus* QQ2 (83), *Bacillus* sp. QQ4 (76), *S. algae* (72.5), and *B. thuringiensis* QQ3 (63). Therefore, the isolates QQ1 and QQ2 could be used as candidate QQ probiotics in aquaculture.

## 4. Materials and Methods

### 4.1. Bacterial Strains and Compounds

*Chromobacterium violaceum* strain CV026 and *Agrobacterium tumefaciens* NTL4 (pZLR4), kindly provided by Prof. Yves Dessaux, Dr. Torabi Delshad, and Dr. Zamani, were used as bioindicators of exogenous AHLs with short and long acyl chains, respectively. AHLs with *N*-acyl side chains from C_4_ to C_8_ in length can induce purple pigment violacein in *C. violaceum* CV026. However, this bioreporter is not able to detect 3-hydroxy-AHLs [67]. With the expression of β-galactosidase activity in the presence of 5-bromo-4-chloro-3-indolyl β-D-galactopyranoside (X-Gal), *A. tumefaciens* NTL4 recognizes substituted (carbonyl or hydroxyl) or unsubstituted AHL-derivatives with side chain lengths from 4 to 12 carbons with the exception of C_4_-AHL [10,67]. The media used for *A. tumefaciens* NTL4 and *C. violaceum* CV026 were included with 50 μg/mL of gentamycin and kanamycin, respectively [42]. *Pseudomonas fluorescence* P3/pME6863 was used as a positive control in AHL degradation assays [26]. When required, PIPES (1,4-piperazinediethanesulfonic acid) buffer was added to the growth media in order to prevent the chemical degradation of the AHLs tested (final pH 6.6). *Vibrio harveyi* PTCC 1755 and *Vibrio alginolyticus* IBRC-M 10,727 were provided by the Persian Type Culture Collection and the Iranian Biological Resource Center, respectively. The synthetic AHLs (Sigma-Aldrich) studied were as follows: *N*-[(RS)-3-Hydroxybutyryl]-l-homoserine lactone (3-oh-C_4_-HSL), *N*-Hexanoyl-l-homoserine lactone (C_6_-HSL), *N*-(β-Ketocaproyl)-l-homoserine lactone (3-oxo-C_6_-HSL), *N*-(3-Oxodecanoyl)-l-homoserine lactone (3-oxo-C_10_-HSL), *N*-(3-Oxotetradecanoyl)-l-homoserine lactone (3-oxo-C_14_-HSL). The signals, together with their producers and detectors, were described in Table 3.

### 4.2. Isolation of Bacteria from the Intestines

Samples were collected during summer 2018 from 30 apparently healthy barramundi fish with sizes ranging from 800 to 2200 g. The fish had been reared in different farms in the South of Iran in cage (Bushehr, 28°21′57.1″ N 51°10′25.5″ E; Hormozgan, 26°45′03.7″ N 54°00′59.3″ E) or earthen pond system (Khuzestan, 30°02′44.4″ N 48°35′25.9″ E). They were transferred on ice to the aquatic animal health clinic, Shahid Chamran University of Ahvaz, Iran. The ventral surface and flank of the fish were disinfected with 70% ethanol, and then the entire intestinal tract was removed and homogenized with sterile 0.89% normal saline (1:10 w/v). The homogenates were serially diluted and 100 µL of dilutions 10^−4^ and 10^−5^ were spread on tryptone soy agar (TSA) plates supplemented with 2% NaCl in triplicates. All plates were incubated for 48 h at 29 °C. The colonies with dissimilar morphologies were selected and sub-cultured for further investigation [51].

### 4.3. Screening for AHL Degrading Bacteria

Minimal agar plates containing nine salts (17.6 g NaCl, 1.47 g Na_2_SO_4_, 0.08 g NaHCO_3_, 0.25 g KCl, 0.04 g KBr, 1.87 MgCl_2_ 6.H_2_O, 0.41 CaCl_2_ 2.H_2_O, 0.008 g SrCl_2_ 6.H_2_O, 0.008 g H_3_BO_3_ per litre) and 1.5% bacteriological agar were prepared. Afterward, the plates were overlaid with the same media containing 10 mg/L mixture of the synthetic AHLs [71]. All selected isolates were streaked and evaluated for growth on the minimal agar plates following incubation at 25 °C for 7–10 days. *Pseudomonas fluorescence* P3/pME6863 was used as a positive control. Given that our objective was the isolation of AHL degrading bacteria incapable of producing any AHLs, the selected isolates were also examined for the production of any exogenous AHLs by cross-feeding assay, as described by Liu et al. (2017) [42]. Each isolate was streaked on Luria–Bertani (LB) agar plates alongside two reporter strains (*C. violaceum* CV026 and *A. tumefaciens* NTL4) and incubated at 28 °C for 48 h. Prior to using *A. tumefaciens* NTL4, the plates were overlaid with a new layer agar containing X-Gal for detection of β-galactosidase activity. The isolates with no potential for induction of the biosensors were separated, sub-cultured, and stored in Tryptic soy broth (TSB) medium containing 30% glycerol for further examination.

### 4.4. Evaluation of AHL Degradation Potential using Synthetic AHLs

Synthetic AHLs were dissolved in ethyl acetate acidified with 1% acetic acid to prepare a stock solution. Prior to studying AHL-degrading activity, different concentrations (0.0025, 0.005, 0.01, 0.025, 0.05, 0.1, 0.25, 0.5, 1, 2.5, 5, and 10 mg/L) were prepared for each AHLs. Ten microlitres from each of these dilutions were separately loaded into the wells produced in the center of buffered LB agar plates (pH 6.6) and seeded with 120 µL overnight culture of the proper biosensor in triplicates. The plates were incubated at 28 °C for 48 h, and the diameter of blue or purple pigmentation of biosensors was measured [18]. Subsequently, a dose-diameter standard curve correlating different concentrations of each AHL and the diameters of colored halos produced by biosensors was calculated. To determine the kinetic of AHL degradation, the well-diffusion method on agar was used. Briefly, each quorum-quenching isolate (QQI) was cultivated overnight to an optical density of 0.25 at 600 nm (OD_600_), and 100 µL from each culture was inoculated into 5 mL buffered (pH 6.6) LB broth previously supplemented with 10 µM of each synthetic AHL in triplicates [19,72]. Buffered LB media with or without the same amount of each AHLs were used as a negative and positive control, respectively. Following incubation at 28 °C and 150 rpm, 2 mL of the cultures were removed and sterilized using a 0.22 µm syringe filter at 24 h and 48 h intervals. 10 µL aliquots of the cell-free media were dispensed into the wells made in LB agar plates overlaid with a lawn of the respective biosensors. Before using *A. tumefaciens* NTL4, a new layer agar containing X-Gal was poured on LB agar plates. The plates were subsequently incubated at 28 °C for 48 h to monitor for the development of a colored halo around the wells, the diameters of the halos were measured and correlated to the corresponding AHL concentration using the dose-diameter standard curve.

### 4.5. Identification of Signal Degrading Isolates

The total genomic DNA was extracted from the screened AHL degrading isolates using the DNeasy Blood and Tissue kit according to the manufacturer’s instructions (Qiagen, Hilden, Germany). PCR amplification of 16S rDNA fragments was carried out using universal primers EUB f933 (5′-GCACAAGCGGTGGAGCATGTGG-3′) and EUB r1387 (5′-GCCCGGGAACGTATTCACCG-3′) or (5′-AGGAGGTGATCCAACCGCA-3′) and RW01 (5′-AACTGGAGGAAGGTGGGGAT-3′) [73,74]. The PCR reactions were performed in a total volume of 25 μL reaction mixture containing 12.5 μL of DreamTaq Green PCR master mix (Thermo Scientific), 0.75 μL of each primer (10 pmol/ul), 2.5 μL of 100 ng/μL sample DNA and 8.5 μL of nuclease-free water. The cycle started with an initial denaturation at 95 °C for 3 min, followed by 40 cycles of denaturation at 95 °C for 25 s, annealing at 60 °C for 30 s and extension at 72 °C for 50 s and a final extension step at 72 °C for five minutes. Five microliters of the amplified products were analyzed by electrophoresis in a 1.2% agarose gel with the amplicon sizes of approximately 400–450 bp. The remaining PCR products were purified using the PCR purification kit following the manufacturer’s protocol (Jena Bioscience, Jena, Germany). The purified PCR products were sequenced and the Basic Local Alignment Search Tool (BLAST) was used to compare resulted sequences with those available in the GenBank repository, and identify their closest homologs. All sequences of the strains were deposited in GenBank.

### 4.6. Sequencing of QQ-Related Genes and Phylogenetic Analysis

Enzymatic degradation of AHLs by QQ bacteria is mainly performed by lactonase and acylase enzymes. Therefore, amplification of the homologs of AHL lactonase gene (*aiiA* and *ytnP*) and AHL acylase gene (*aaC*) by PCR was examined in selected quorum quenching isolates (QQIs). The *aiiA* homolog genes were amplified using primers AIF (5′-TAAATGTAAAGGTGGATACATAATGACAGT-3′) and AIR (5′-AGCTCATGACTTTTTGCACTATATATA-3′) [72]. The new primers of YtnPF (5′-CGCCTGCCATTTCTGCTTTT-3′) and YtnPR (5′-AATTGGGTACGGCAAGCTGA-3′) as well as aacSh.aF (5′-GCCTACAAGGCACTCAAGGT-3′) and aacSh.aR (5′-GCGCTTCGACTATAGGTCCC-3′) were designed for partial amplification of *ytnP* and *aaC* homolog genes, respectively [19,35]. PCR conditions for amplification of homologs of the lactonase gene started with a denaturation step at 94 °C for 4 min followed by 30 cycles at 94 °C for 30 s, 55 °C for 30 s, 72 °C for 1 min and a final extension at 72 °C for 7 min. The *aaC* homolog gene was amplified using the following parameters: initial denaturation at 94 °C, 3 min; 30 cycles of 94 °C, 30 s; 55 °C, 30 s; and 74 °C, 5 min, with a final extension at 72 °C for 5 min. Following the sequencing of the purified PCR products as described above, the sequences obtained were compared to those of known lactonases and acylases already present in the NCBI database using BLAST. The multiple amino acid sequence alignment of the putative AHL degrading enzymes with other AHL lactonase and acylase homologs was conducted using the ClustalW algorithm in the CLC Main Workbench software version 8 [26]. The phylogenetic tree was constructed using the neighbor-joining method with 1000 bootstrap replicates in the MEGA X software [75].

### 4.7. Antagonistic Activity in AHL Degrading Bacteria

Direct and extracellular antagonistic activities of the selected isolates were investigated against *V. harveyi* and *V. alginolyticus* using spot on the lawn and agar well diffusion methods, respectively. In order to determine direct antibacterial activity, 15 mL of an overnight culture of both pathogenic bacteria were centrifuged at 12,000× *g* for 10 min, and the pellet was resuspended in sterile phosphate saline buffer (PBS) and adjusted to an OD_600_ of 0.13. 100 µL of each suspension were spread on TSA plates supplemented with 2% NaCl. TSA plates were kept at the laminar flow for 10 min, and then 5 µl of an overnight culture of AHL-degrading isolates (OD_600_ 0.25) was spotted on the surface of inoculated TSA plates. The plates were incubated for 48 h at 26 °C for visible inhibition zones around the spotted cultures [76]. Extracellular antibacterial activity of QQIs was tested on TSA plates. Bacterial cultures grown in TSB medium containing 2% NaCl for 24 h were centrifuged at 10,000× *g* for 12 min, and subsequently filter-sterilized using 0.22 µm syringe filters. Fifty µL of the supernatants were loaded into 6 mm wells in TSA plates inoculated with 100 µL aliquot of suspensions *V. harveyi* or *V. alginolyticus* (OD_600_ 0.13). The plates were incubated for 48 h at 26 °C. Growth inhibition zones around the wells were measured after the incubation period. One-hundred microliters of sterile TSB was used as a negative control [77].

### 4.8. Detection of AHL in Tested Vibrio harveyi and Vibrio alginolyticus

To qualitatively explore the range of AHLs produced by *V. harveyi* and *V. alginolyticus*, these bacteria were cross-fed alongside the biosensors *C. violaceum* CV026 and *A. tumefaciens* NTL4 as described by Liu et al. (2017) [42]. In order to extract the AHLs, *V. harveyi* and *V. alginolyticus* were grown in TSB medium supplemented with 2% NaCl to stationary phase (OD_600_ 2.7). Fifty mL of the whole cultures were mixed with the same volume of 1% acidified ethyl acetate. The mixtures were vigorously shaken, and the phases were allowed to separate. The upper organic phase, including the AHL fractions was removed. The extraction operation was repeated twice. The pooled ethyl acetate portions were evaporated and dried using a rotary evaporator. The residues were dissolved in 100 µL of ethyl acetate and were stored at −20 °C until use. The obtained crude extracts were evaluated by agar well diffusion method using two biosensors. Briefly, 15 µL of the crude extracts of *V. harveyi* and *V. alginolyticus* were placed into the wells produced in LB agar seeded with the biosensors. The Petri plates were incubated at 28 °C for 48 h to monitor for the production of a halo of the appropriate color around the wells [78].

### 4.9. In vitro Assay of QQ Activity on Vibrio harveyi and Vibrio alginolyticus’ AHLs

The QQ potentials of the selected probiotic isolates against crude extracts extracted from *V. harveyi* and *V. alginolyticus* were tested, as described by Mahmoudi et al. (2011) [79]. Briefly, 10 µL of the concentrated extracts were incorporated into 1000 µL of buffered LB broth inoculated with an overnight culture of QQ bacteria in triplicates followed by incubation at 25 °C for 24 h and 150 rpm. The same volume of the extracts was incubated with buffered LB broth as negative control. The AHLs remaining from all replications were extracted with ethylacetate, dried, and resuspended in 20 µL of the same solvent. The presence of the AHL in the extracts (15 µL) was initially detected by the agar well-diffusion test using *A. tumefaciens* NTL4 as detectors. To confirm the QQ activity, 20 µL of the extracts were spotted on a TLC plate (TLC Silica gel 60 RP-18 F_254_S, Merck, Germany) as stationary phase and developed in 60% methanol (v/v) as mobile phase until the solvent front line reached to 1.5 cm from top edge of the plate. The plates were completely dried and each plate was held in a large Petri dish (150 mm × 150 mm) before being overlaid with a thin layer agar (3 mm) containing *A. tumefaciens* NTL4 and incubated at 28 °C for five days. To prepare *A. tumefaciens* NTL4 overlay, an overnight culture of *A. tumefaciens* NTL4 in LB broth medium supplemented with gentamycin was mixed with equal volume of LB broth containing gentamycin, 1.5% agar (0.75% final agar) and 50 µg/mL X-Gal. The synthetic AHLs prepared in ethylacetate were used as reference standards. The recorded retention factors (R_f_) were compared to those of standards to determine the approximate type of extracted AHLs as well as their possible inactivation by QQIs.

### 4.10. Location of AHL degrading Enzymes in Selected Bacteria

To determine if the AHL degradation activity was associated with extracellular or cell-bound location, AHL degrading bacteria were cultured in buffered TSB supplemented with 2% NaCl at 28 °C for 24 h. The cultures were centrifuged at 12,000× *g* for 12 min, and the supernatants were sterilized using 0.22 µm syringe filters. Synthetic AHLs (3-oh-C_4_-HSL, C_6_-HSL, 3-oxo-C_6_-HSL, 3-oxo-C_10_-HSL, and 3-oxo-C_14_-HSL) in a final concentration of 10 µM were added to 2.5 mL of supernatants followed by incubation at 28 °C for 24 h and 120 rpm. The agar well-diffusion test was used for the detection of the remaining AHLs, as previously described [19,26].

### 4.11. In vitro Tests of Candidate Probiotic Bacteria

#### 4.11.1. Enzyme Production and Spore Formation

The selected isolates were qualitatively screened for the production of extracellular proteinase, lipase, amylase, and phytase. They were streaked on different media at 28 °C for 72 h in three replicates, and proteolytic activity was assayed using skim milk agar media [80]. The appearance of transparent halos around the streaked bacteria interpreted as positive protease activity. Lipolytic activity was tested on T medium containing 1% tryptone, 1 mM CaCl_2_, 1% tween 80, and 1.2% agar [81]. An opaque halo around the cultured bacteria due to crystals of the calcium salt of the fatty acids liberated by lipolysis was regarded as a positive lipase activity. The presence of amylolytic activity was determined by starch medium consisting of 12 g starch, 4 g peptone, and 12 g agar per liter [82]. The plates were then covered with a 1% Lugol’s iodine solution for the detection of a clear halo in a dark field, representing a positive activity. Phytase screening media were prepared as follows: 15 g glucose, 1 g sodium phytate, 2 g NH_4_NO_3_, 0.5 g KCl, 0.5 g MgSO_4_ 7.H_2_O, 0.3 g MnSO_4_, 0.3 g FeSO_4_ 7.H_2_O and 20 g agar per liter (final pH 7.5). Bacterial isolates able to hydrolyze sodium phytate were distinguished by their surrounding clear halo [83]. In order to identify the potential of the isolates for sporulation, they were cultured on spore preparation agar medium (3.3 g peptone, 1 g beef extract powder, 5 g NaCl, 2 g K_2_HPO_4_, 1 g KCl, 0.25 g MgSO_4_. 7.H_2_O, 0.01 g MnSO_4_, 5 g glucose and 15 g agar per liter) and were incubated at 28 °C for 5–7 days [84]. Subsequently, each isolate was stained using a gram staining commercial kit and was observed under a light microscope for the possible production of spores.

#### 4.11.2. Resistance to Bile Salts and pH

Bacterial cultures were grown in TSB medium at 28 °C overnight (18 h), pelleted down by centrifugation (10,000× *g*, 5 min), washed twice with sterile PBS buffer (pH 7.1) and resuspended in similar buffer to achieve an OD_600_ of 0.66. One-hundred microliters of the suspension were incorporated either to 900 µL of suspensions containing a similar ratio (1:1) of two bile salts (sodium cholate and sodium deoxycholate) or 900 µL of sterile PBS solutions pre-adjusted to various degrees of pH (1.5, 3, 4.5, 6, 7, and 9) using HCl 1 M. Following incubation at 30 °C for 1.5 h, the bacterial suspensions were serially diluted in PBS buffer, and their abundance was evaluated in terms of viable colony counts in triplicates [55].

#### 4.11.3. Growth at Different Salinities

Fifty microliters of the bacterial suspensions prepared as described above were utilized to inoculate in TSB medium supplemented with different concentrations of NaCl (0, 2, 4, 8%) in triplicates. Following incubation for 0 and 24 h, 150 µL aliquots of the media were loaded into 96-well microplates, and then OD_630_ was measured in each well [85].

#### 4.11.4. Hydrophobicity

To investigate surface hydrophobicity, bacterial suspensions obtained, as previously described were adjusted to an OD_600_ of 1.0. Then, 5 mL of bacterial suspensions were mixed with 1 mL of toluene by vigorous vortexing for 1 min, allowing the separation of the aqueous and organic phases for 1 h at room temperature. Following phase separation, the final OD of aqueous phase was determined. The cell surface hydrophobicity was calculated using the following formula: Surface hydrophobicity (%) = ([A_0_ − A_1_]/A_0_) × 100, where A_1_ and A_0_ are the ODs at the aqueous phase and the original suspension respectively. The assay was performed in triplicate [86].

#### 4.11.5. Auto-Aggregation and Co-Aggregation

Cellular auto-aggregation was assayed following the method proposed by Meidong et al. (2017) with slight modifications [57]. Five mL of each bacterial suspensions in separate glass tubes were adjusted to an OD_600_ of 1.0, as previously described. They were incubated at room temperature in an undisturbed manner for 5 h, and the optical density of upper suspension was measured at 600 nm. The percentage of auto-aggregation was calculated by the following equation, where A_0_ and A_t_ represent the OD_600_ after 0 and 5 h incubation. The experiments were performed in three independent replications: Auto-aggregation (%) = 1 − (A_t_/A_0_) × 100.

Co-aggregation of different isolates with marine fish pathogens, *V. harveyi* and *V. alginolyticus*, was assessed as described by Handley et al. (1987) with modification [87]. Briefly, a suspension (4 mL) of each strain together with a suspension (4 mL) of *V. harveyi* or *V. alginolyticus* (both at an OD_600_ of 1.0) was placed in a glass tube, mixed vigorously and immediately transferred into a spectrophotometry cuvette (1.5 mL volume). Control tubes included 4 mL of individual bacterial suspensions. The OD_600_ of bacterial suspensions was measured following incubation at room temperature for 2 h and 24 h. The percentage of co-aggregation was calculated using the following equation, where A represents OD_600_, QQ, and P represent individual pathogen or QQIs in the control tubes and (QQ+P) their mixture. The tests were performed in three independent replications: Co-aggregation (%) = {[(A_QQ_ + A_P_)/2] − A(_QQ+P_)}/[(A_QQ_ + A_P_)/2] × 100.

#### 4.11.6. Adherence to Intestinal Mucus

Intestinal mucus was collected by gently scraping off the inner intestinal surfaces from five 150-gr healthy barrumudi fish starved for 48 h. The rinsed mucus in 10 mM PBS (pH 7.2) was centrifuged at 10,000× *g* for 15 min at 4 °C, and the supernatants were sterilized using 0.22 µm syringe filters. The protein concentration of the mucus was determined and adjusted to 0.5 mg/mL in PBS as described in the commercial kit (Pars Azmoon kit, Tehran, Iran). The adjusted mucus suspensions were aliquoted and stored at −20 °C until use. The potential of the isolates to adhere to intestinal mucus was tested by the crystal violet method, as described by Geraylou et al. (2012) [62]. The experiment was performed in sterile 96-well microplates together with six replications for each isolate. The wells were coated with 150 µL of intestinal mucus at 4 °C for 24 h, and then unattached mucus was removed, and the wells were washed twice with sterile PBS. 100 µL of each isolate previously adjusted to an OD_600_ of 0.6 (A_0_) were placed into the wells and incubated for 3 h at room temperature. Afterward, non-adherent bacteria were washed away with sterile PBS, bound bacteria were fixed for 25 min at 60 °C and stained with 100 µL filtered crystal violet (0.1%) for 45 min, and the wells were washed three times with sterile PBS. The stain attached to bacteria was solubilized using 100 µL of citrate buffer (20 mM, pH 4.3). Stained mucus without added bacteria was considered as a negative control. The absorbance of released suspension for tested bacteria or negative control was read in a microplate reader at 600 nm wavelength (A_1_, A_control_). The percentage of bacterial adhesion to mucus was calculated as follows: Adhesion to mucus (%) = (A_1_ − A_control_)/A_0_ × 100.

#### 4.11.7. Growth on Intestinal Mucus

The potential of the bacteria to grow in the gut was monitored by sterile 96-well microplates. Briefly, twenty microliters of the bacterial suspensions prepared as described before (OD_600_ 1.0), were inoculated in triplicate into the wells pre-pipetted with 250 µL of sterile diluted mucus (as previously obtained) or TSB media supplemented with 2% NaCl. The wells containing only mucus or TSB were used as control. During incubation of the plate at 25 °C for 48 h, the optical density was recorded every 2 h at 640 nm. To calculate specific growth rate (µ), the exponential growth phase followed by the linear regression of the log2-transformed data was used as proposed by Geraylou et al. (2012) [62]. Doubling time (t_d_) was calculated according to the formula described by Vine et al. (2004) [63]. Finally, the specific growth rate and doubling time of the candidate bacteria were compared in mucus and TSB medium using the following equation: Doubling time (t_d_) = ln 2/µ.

#### 4.11.8. Antibiotic Resistance Test

Antibiotic susceptibility of the selected strains to 9 antibiotics, selected because they represent an important family of antibiotics or because they are commonly applied in aquaculture settings, was determined using the disc diffusion method. Antibiotic discs of doxycycline (30 µg), oxytetracycline (30 µg), erythromycin (15 µg), gentamicin (10 µg), enrofloxacin (5 µg), florfenicol (30 µg), flumequine (30 µg), oxolinic acid (2 µg) and trimethoprim/sulfamethoxazole (25 µg) were placed on the Mueller-Hinton agar plates seeded with 100 µL overnight culture of each QQ strain (McFarland standard 0.5). Following incubation at 37 °C for 24 h, the zone of growth inhibition was measured, and the antibiotic sensitivity was determined based on their activity [84].

#### 4.11.9. Safety of the Selected Bacteria for Fish

To ascertain the possible pathogenicity of the isolated QQ bacteria, 100 µL of adjusted suspension (10^8^ CFU/mL) of the strains were injected intraperitoneally into two separate groups of ten barramundi fish with an average size of 30 g. The control groups were injected with the same volume of sterile saline (0.89%). All groups were maintained in fiberglass tanks supplied with UV sterilized running marine water for two weeks. Fish were monitored daily to trace any adverse clinical signs of disease followed by the microbiological examination of hematopoietic organs at the end of the experiment [53,88]. This study was approved by the institutional ethics committee of the Shahid Chamran University of Ahvaz, Iran, under approval number EE/98.24.3.71483/scu.ac.ir. All procedures on animals in this experiment were carried out according to the guide for the care and use of laboratory animals by the National Academy of Sciences (National Institutes of Health publication No. 86–23) [89].

### 4.12. Statistical Analysis

All data presented in the current study were expressed as mean ± standard deviation. Statistical analysis was carried out using SPSS program version 22. The normality of the data was checked by the Shapiro–Wilk test. Student’s t-test and one-way analysis of variance (ANOVA) followed by Tukey’s multiple-comparison test were performed to determine significant differences between specific groups. Differences were considered statistically significant at the 5% probability level. A scoring system was established to evaluate the suitability of the probiotic stains based on the statistical analyses, as well as pre-determined ranges (Table 2 and Supplementary Table S6). This allowed to select the most potent QQ probionts.

## 5. Conclusions

In conclusion, several autochthonous AHL degrading bacteria, capable of inactivating synthetic and natural AHLs from several pathogenic *Vibrio* spp. were isolated from the intestine of Asian seabass. The profile of these isolates in terms of QQ activity and their probiotic activity were investigated. In vitro investigation allowed the strains, in particular QQ1 and QQ2, to show high potential as QQ probionts. Further studies in vivo are required to confirm their beneficial effects and also their protection against marine vibriosis. This study represents the first evidence of the presence of beneficial QQ probiotics in *L. calcarifer*.

## Figures and Tables

**Table 1:** Identification of quorum quenching bacteria isolated from the intestinal tract of barramundi fish.

Isolate Number	Closest Known Species (16S rDNA Sequence)	Identity (%)	Location-Culture System	Accession Number
QQ1	*Bacillus thuringiensis*	99.76	Bushehr-cage culture	SAMN13108109
QQ2	*Bacillus cereus*	99.72	Hormozgan-cage culture	SAMN13108110
QQ3	*Bacillus thuringiensis*	100	Hormozgan-cage culture	SAMN13108111
QQ4	*Bacillus* sp.	99.29	Bushehr-cage culture	SAMN13108112
QQ5	*Shewanella algae*	99.08	Khuzestan-earthen pond	SAMN13108113
QQ6	*Carnobacterium maltaromaticum*	97.41	Bushehr-cage culture	SAMN13108114
QQ7	*Bacillus* sp.	96.86	Bushehr-cage culture	SAMN13108115
QQ8	*Bacillus* sp.	96.63	Bushehr-cage culture	SAMN13108116
QQ9	*Bacillus* sp.	95.35	Hormozgan-cage culture	SAMN13108117
QQ10	*Bacillus* sp.	96.35	Hormozgan-cage culture	SAMN13108118

**Table 2:** Production of extracellular enzymes and spores by selected quorum quenching bacteria.

Isolates	Enzyme Production				Spore Formation	Total Score
	Protease	Lipase	Amylase	Phytase		
QQ1	++	++	++	−	+	7
QQ2	+++	+	-	−	+	5
QQ3	+	−	++	+	+	5
QQ4	+++	+	−	+	+	6
QQ5	+++	++	−	−	−	5

Potent activity (>1 cm): +++; Moderate activity (0.5–1 cm): ++; Partial activity (<0.5 cm): +; No activity: −.

**Table 3:** The studied synthetic AHLs in combination with the signal producers and detectors.

Signals (AHLs)	Pathogens	AHL Detector	References
3-oh-C_4_-HSL	*Vibrio harveyi*	*A. tumefaciens* NTL4	[68]
	*Vibrio campbelli*		
	*Vibrio parahaemolyticus*		
3-oh-C_4_-HSL	*Vibrio alginolyticus*	*A. tumefaciens* NTL4	[42]
3-oxo-C_10_-HSL		*A. tumefaciens* NTL4	
3-oxo-C_14_-HSL		*A. tumefaciens* NTL4	
C_6_-HSL	*Vibrio anguillarum*	*C. violaceum* CV026	[69]
3-oxo-C_10_-HSL		*A. tumefaciens* NTL4	
C_6_-HSL	*Vibrio salmonicida*	*C. violaceum* CV026	[70]
3-oxo-C_6_-HSL		*C. violaceum* CV026	

## Supplementary Materials

The following are available online at https://www.mdpi.com/1660-3397/18/1/23/s1, Table S1: Resistance of quorum quenching bacteria to bile salts, Table S2: Survival of quorum quenching bacteria in various degrees of pH, Table S3: Growth of quorum quenching bacteria in different salinities, Table S4: Co-aggregation percentage of quorum quenching bacteria, Table S5: Specific growth rate and doubling time of quorum quenching bacteria in marine broth and mucus, Table S6: Antibiotic resistance of quorum quenching bacteria.
