# Supplementary material for: Quorum Quenching Properties and Probiotic Potentials of Intestinal Associated Bacteria in Asian Sea Bass Lates calcarifer
Source: Mar Drugs. 2019 Dec 26;18(1):23. doi: 10.3390/md18010023 (PMC7024293; doi:10.3390/md18010023)
Supplement: Supplementary file 1 [file marinedrugs-18-00023-s001.pdf]

Supplementary Materials:

Quorum quenching properties and probiotic potentials of intestinal associated bacteria in Asian sea bass *Lates calcarifer*

Reza Ghanei-Motlagh [1,3], Takavar Mohammadian [1,*], Dariush Gharibi [2], Simon Menanteau-Ledouble [3,*], Esmaeil Mahmoudi [4], Mohammad Khosravi [2], Mojtaba Zarea [1] and Mansour El-Matbouli [3]

[1] Department of Clinical Sciences, Faculty of Veterinary Medicine, Shahid Chamran University of Ahvaz, Ahvaz, Iran

[2] Department of Pathobiology, Faculty of Veterinary Medicine, Shahid Chamran University of Ahvaz, Ahvaz, Iran

[3] Clinical Division of Fish Medicine, University of Veterinary Medicine, Vienna, Austria

[4] Department of Plant Protection, Faculty of Agriculture, Isfahan (Khorasgan) Branch, Islamic Azad University, Isfahan, Iran

* Correspondence: t.mohammadian@scu.ac.ir (T.M.); Simon.Menanteau-Ledouble@vetmeduni.ac.at (S.M.-L.)

Supplementary Table S1. Resistance of quorum quenching bacteria to bile salts.

	Bile concentration			
Isolates	2.5%	5%	7.5%	Total score
QQ1	8.00 ± 0.01[a]	7.97 ± 0.01[a]	7.90 ± 0.01[a]	12
QQ2	7.97 ± 0.04[a]	7.96 ± 0.02[a]	7.94 ± 0.03[a]	12
QQ3	7.88 ± 0.02[b]	7.85 ± 0.01[b]	7.74 ± 0.01[b]	9
QQ4	7.97 ± 0.01[a]	7.94 ± 0.01[a]	7.92 ± 0.02[a]	12
QQ5	7.97 ± 0.02[a]	7.94 ± 0.01[a]	7.91 ± 0.02[a]	12

Data are expressed as log cfu/ml (Mean ± SD) (n=3). Different superscript letters in each column indicate statically significant differences between values ($P < 0.05$). The values were rated as: a (4), b (3)

Supplementary Table S2. Survival of quorum quenching bacteria in various degrees of pH.

			pH			Total
Isolates	1.5	3	4.5	7	9	score
QQ1	7.66 ± 0.05[a]	7.85 ± 0.01[a]	7.96 ± 0.01[a]	8.05 ± 0.02[a]	8.01 ± 0.02[a]	20
QQ2	7.64 ± 0.01[a]	7.86 ± 0.01[a]	7.96 ± 0.01[a]	7.98 ± 0.03[b]	7.98 ± 0.03[a]	19
QQ3	7.31 ± 0.06[b]	7.77 ± 0.04[b]	7.87 ± 0.03[b]	7.89 ± 0.01[c]	7.89 ± 0.01[b]	14
QQ4	7.67 ± 0.02[a]	7.87 ± 0.02[a]	7.97 ± 0.02[a]	7.98 ± 0.03[b]	7.96 ± 0.02[a]	19
QQ5	ND	ND	7.68 ± 0.02[c]	8.01 ± 0.03[ab]	8.00 ± 0.02[a]	9.5

Data represent the log cfu/ml (Mean ± SD) of three replicates. A different superscript within the same column indicates significant difference among values ($P < 0.05$). The values were rated as follow: a (4), ab (3.5), b (3), bc (2.5), c (2) Not detected (ND).

Supplementary Table S3. Growth of quorum quenching bacteria in different salinities.

		Salinity (OD)			Total
Isolates	0%	2%	4%	8%	score
QQ1	0.456 ± 0.01[b]	0.517 ± 0.01[c]	0.362 ± 0.01[b]	0.181 ± 0.01[a]	12
QQ2	0.428 ± 0.01[b]	0.690 ± 0.01[a]	0.333 ± 0.01[c]	0.130 ± 0.01[b]	12
QQ3	0.215 ± 0.01[d]	0.346 ± 0.01[e]	0.328 ± 0.01[cd]	0.079 ± 0.01[c]	4.5
QQ4	0.381 ± 0.01[c]	0.458 ± 0.01[d]	0.307 ± 0.01[d]	0.120 ± 0.01[b]	5
QQ5	0.620 ± 0.01[a]	0.541 ± 0.01[b]	0.487 ± 0.01[a]	0.115 ± 0.01[b]	14

Values (Mean ± SD, n=3) with various lowercase letters in a column indicate significant differences ($P < 0.05$). The data were scored as: a (4), ab (3.5), b (3), bc (2.5), c (2), cd (1.5), d (1), de (0.5), e (0).

Supplementary Table S4. Co-aggregation percentage of quorum quenching bacteria.

	**Coaggregation (%)**				Total score
	Vibrio harveyi		*Vibrio alginolyticus*		
Isolates	2 h	24 h	2 h	24 h	
QQ1	11.74 ± 2.00^{bc}	33.66 ± 1.82^{a}	22.25 ± 3.25^{b}	41.34 ± 1.95^{b}	12.5
QQ2	16.91 ± 0.72^{a}	23.59 ± 1.75^{b}	30.58 ± 2.98^{a}	52.71 ± 3.13^{a}	15
QQ3	13.17 ± 0.33^{ab}	23.62 ± 1.18^{b}	16.59 ± 1.18^{bc}	37.26 ± 1.60^{b}	12
QQ4	15.88 ± 2.10^{a}	24.87 ± 1.83^{b}	21.20 ± 0.40^{b}	36.58 ± 0.96^{b}	13
QQ5	8.40 ± 1.26^{c}	16.77 ± 1.48^{c}	12.13 ± 2.76^{c}	22.96 ± 0.76^{c}	8

The results indicate mean ± standard deviation of three replicates at two intervals. Values with the same letter within columns are not statistically significant ($P < 0.05$). Data were rated as: a (4), ab (3.5), b (3), bc (2.5), c (2), cd (1.5), d (1).

Supplementary Table S5. Specific growth rate and doubling time of quorum quenching bacteria in marine broth and mucus.

Isolates	**Specific growth rate (μ)**	**Doubling time (t_d)**	**r^2**	Total score
**Marine broth**				
QQ1	0.291 ± 0.037^{a}	0.85 ± 0.06^{a}	0.94 ± 0.01	8
QQ2	0.177 ± 0.008^{b}	1.22 ± 0.08^{b}	0.93 ± 0.01	6
QQ3	0.184 ± 0.023^{b}	1.04 ± 0.06^{ab}	0.96 ± 0.01	6.5
QQ4	0.180 ± 0.014^{b}	1.24 ± 0.07^{b}	0.95 ± 0.02	6
QQ5	0.197 ± 0.011^{b}	1.00 ± 0.10^{a}	0.95 ± 0.03	7
**Mucus**				
QQ1	0.074 ± 0.006^{b}	3.08 ± 0.17^{c}	0.96 ± 0.01	5
QQ2	0.085 ± 0.005^{b}	2.40 ± 0.10^{b}	0.93 ± 0.02	6
QQ3	0.062 ± 0.003^{b}	3.63 ± 0.15^{d}	0.94 ± 0.03	4
QQ4	0.065 ± 0.009^{b}	2.67 ± 0.12^{b}	0.92 ± 0.02	6
QQ5	0.133 ± 0.015^{a}	0.93 ± 0.06^{a}	0.94 ± 0.02	8

Values (Mean ± SD, n=3) in the same column sharing identical superscripts are not significantly different ($P < 0.05$). Rating to the isolates (for SGR) was performed according to the respective differences: a (4), ab (3.5), b (3), bc (2.5), c (2), cd (1.5), d (1).

Supplementary Table S6. Antibiotic resistance of quorum quenching bacteria.

**Antibiotics**	QQ1	QQ2	QQ3	QQ4	QQ5
Doxycycline (D, 30 μg)	S	S	S	S	S
Oxytetracycline (OT, 30 μg)	S	S	S	S	S
Erythromycin (E, 15 μg)	S	S	S	S	S
Gentamicin (CN, 10 μg)	S	S	S	S	S
Enrofloxacin (ENR, 5 μg)	S	S	S	S	S
Florfenicol (FF, 30 μg)	S	S	S	S	S
Flumequine (UB, 30 μg)	S	S	S	S	S
Oxolinic acid (OA, 2 μg)	S	S	S	S	S
Trimethoprim/Sulfamethoxazole (SXT, 25 μg)	R	R	R	S	S
Total score	8	8	8	9	9

Susceptible (S): 1, Resistant (R): 0. Susceptibility of the isolates to tested antibiotics was regarded as a positive score.
